# Do nuclear magnetic resonance (NMR)-based metabolomics improve the prediction of pregnancy-related disorders? Findings from a UK birth cohort with independent validation

**DOI:** 10.1186/s12916-020-01819-z

**Published:** 2020-11-23

**Authors:** Nancy McBride, Paul Yousefi, Sara L. White, Lucilla Poston, Diane Farrar, Naveed Sattar, Scott M. Nelson, John Wright, Dan Mason, Matthew Suderman, Caroline Relton, Deborah A. Lawlor

**Affiliations:** 1 MRC Integrative Epidemiology Unit at the University of Bristol, Bristol, UK; 2 NIHR Bristol Biomedical Research Centre, University of Bristol, Bristol, UK; 3 Population Health Sciences, University of Bristol, Bristol, UK; 4 Department of Women and Children’s Health, Faculty of Life Sciences and Medicine, King’s College London, London, UK; 5 Bradford Institute for Health Research, Bradford Teaching Hospitals NHS Foundation Trust, Bradford, UK; 6 Cardiovascular and Medical Sciences, British Heart Foundation Glasgow, Cardiovascular Research Centre, University of Glasgow, Glasgow, UK; 7 School of Medicine, University of Glasgow, Glasgow, UK

**Keywords:** Prediction, Pregnancy, Metabolomics

## Abstract

**Background:**

Prediction of pregnancy-related disorders is usually done based on established and easily measured risk factors. Recent advances in metabolomics may provide earlier and more accurate prediction of women at risk of pregnancy-related disorders.

**Methods:**

We used data collected from women in the Born in Bradford (BiB; *n* = 8212) and UK Pregnancies Better Eating and Activity Trial (UPBEAT; *n* = 859) studies to create and validate prediction models for pregnancy-related disorders. These were gestational diabetes mellitus (GDM), hypertensive disorders of pregnancy (HDP), small for gestational age (SGA), large for gestational age (LGA) and preterm birth (PTB). We used ten-fold cross-validation and penalised regression to create prediction models. We compared the predictive performance of (1) risk factors (maternal age, pregnancy smoking, body mass index (BMI), ethnicity and parity) to (2) nuclear magnetic resonance-derived metabolites (*N* = 156 quantified metabolites, collected at 24–28 weeks gestation) and (3) combined risk factors and metabolites. The multi-ethnic BiB cohort was used for training and testing the models, with independent validation conducted in UPBEAT, a multi-ethnic study of obese pregnant women.

**Results:**

Maternal age, pregnancy smoking, BMI, ethnicity and parity were retained in the combined risk factor and metabolite models for all outcomes apart from PTB, which did not include maternal age. In addition, 147, 33, 96, 51 and 14 of the 156 metabolite traits were retained in the combined risk factor and metabolite model for GDM, HDP, SGA, LGA and PTB, respectively. These include cholesterol and triglycerides in very low-density lipoproteins (VLDL) in the models predicting GDM, HDP, SGA and LGA, and monounsaturated fatty acids (MUFA), ratios of MUFA to omega 3 fatty acids and total fatty acids, and a ratio of apolipoprotein B to apolipoprotein A-1 (APOA:APOB1) were retained predictors for GDM and LGA. In BiB, discrimination for GDM, HDP, LGA and SGA was improved in the combined risk factors and metabolites models. Risk factor area under the curve (AUC 95% confidence interval (CI)): GDM (0.69 (0.64, 0.73)), HDP (0.74 (0.70, 0.78)) and LGA (0.71 (0.66, 0.75)), and SGA (0.59 (0.56, 0.63)). Combined risk factor and metabolite models AUC 95% (CI): GDM (0.78 (0.74, 0.81)), HDP (0.76 (0.73, 0.79)) and LGA (0.75 (0.70, 0.79)), and SGA (0.66 (0.63, 0.70)). For GDM, HDP and LGA, but not SGA, calibration was good for a combined risk factor and metabolite model. Prediction of PTB was poor for all models. Independent validation in UPBEAT at 24–28 weeks and 15–18 weeks gestation confirmed similar patterns of results, but AUCs were attenuated.

**Conclusions:**

Our results suggest a combined risk factor and metabolite model improves prediction of GDM, HDP and LGA, and SGA, when compared to risk factors alone. They also highlight the difficulty of predicting PTB, with all models performing poorly.

## Background

Around 40% of all pregnancies are complicated by one or more of gestational diabetes mellitus (GDM), hypertensive disorders of pregnancy (HDP), small or large for gestational age (SGA, LGA) and preterm birth (PTB). These pregnancy-related disorders have adverse short- and long-term consequences for the mother and child [1–7]. Established risk factors for pregnancy-related disorders include pregnancy smoking, maternal age, body mass index (BMI), maternal ethnicity and parity [6, 8–12]. However, a large proportion of disorders occur in women without any known risk factors. Current identification of women who are ‘high-risk’ uses clinical screening of these risk factors, sometimes in combination with early pregnancy measures of glucose for GDM [13], blood pressure for HDP [6], ultrasound for SGA and LGA [14] and cervical length measurement/fetal fibronectin for PTB [15]. However, whilst glucose measures in early pregnancy can identify women with undiagnosed existing diabetes, neither it nor established risk factors in early pregnancy predict GDM risk accurately [16]. Ultrasound has poor consistency, is prone to human error and often fails to identify SGA or LGA babies until very late in pregnancy [17]. Cervical length and fetal fibronectin have improved the prediction of PTB but are invasive and only predict ‘imminent’ preterm birth in women where this is suspected [15].

These pregnancy-related disorders often co-occur, with women with GDM more likely to have pregnancies complicated by hypertension or pre-eclampsia (PE), and their offspring being born LGA [2]. Similarly, women with HDP are more likely to have their offspring born SGA or preterm [5]. However, most research focuses on single outcomes. This multimorbidity should be addressed to see if a common prediction tool, or a tool with an overlap of variables, can be developed for predicting global risk of several pregnancy-related disorders. It may also enable identification of women likely to have a healthy pregnancy [18–20].

Metabolomics might improve prediction of pregnancy-related disorders. Metabolite levels are known to change markedly during pregnancy [21, 22] and associate with cardio-metabolic outcomes (known correlates of pregnancy-related disorders) [18] and with pregnancy-related disorders in some studies [23]. Most studies exploring the value of metabolomics in predicting pregnancy-related disorders have focused on GDM, PE or SGA. The most notable omics predictor that has been identified to date is soluble fms-like tyrosine kinase 1 (sFlt-1) and placental growth factor (PlGF) ratio for predicting PE. sFlt-1:PlGF is an accurate predictor of PE in both low- and high-risk pregnant women [24]. With respect to metabolite prediction, two studies reported excellent predictive discrimination for SGA (area under the curve - AUC > 0.90)—one study which developed a metabolomic model of five metabolites [25] and another of 19 metabolites [26]. However, these were based on small samples of 83 and 8 women, respectively. Similarly, a study reported that a panel of four mass spectrometry-derived metabolites could predict spontaneous PTB with a partial AUC (i.e. an alternative to AUC, whereby only the regions of ROC space where data are observed are included) of 12.6 in 105 women [27]. These studies did not compare their models to the existing risk factors or undertake external validation. A systematic review of metabolomic prediction of SGA identified 15 studies [28]. Of these, only three were designed for prediction purposes and provided any metric of prediction. Two of these three had sample sizes of 80 and 83 women. None of them sought external validation. For GDM, nuclear magnetic resonance (NMR)-derived metabolites have been found to distinguish between women who did and did not go on to develop GDM, when looked at in early pregnancy. However, discrimination did not improve when added to a risk prediction model of candidate biomarkers [29].

A recent collaboration between the Pregnancy Outcomes Prediction study (POPs) and the Born in Bradford (BiB) cohort (the latter used as external validation) using mass spectrometry metabolomics (> 1100 semi-quantified untargeted metabolites) has shown that 4-hydroxyglutamate improves prediction of PE over risk factors alone [30]. The same collaboration found that sFlt-1:PlGF and a ratio of combining four metabolites (1-(1-enyl-stearoyl)-2-oleoyl-GPC, 1,5-anhydroglucitol,5α-androstan-3α,17α-diol disulfate and N1,N12-diacetylspermine) is a better predictor of fetal growth restriction than sFlt-1:PlGF combined with risk factors [31].

In this study, our aim was to see whether NMR-derived metabolites could improve the prediction of pregnancy-related disorders, over and above established risk factors (pregnancy smoking, maternal age, BMI, maternal ethnicity and parity). We focused on the prediction of five common pregnancy-related disorders: GDM, HDP, SGA, LGA and PTB. We used two samples, (1) women in the BiB cohort, used for training and testing the prediction models, and (2) obese pregnant women (BMI ≥ 30 kg/m^2^) in the UPBEAT study, used for external validation of the prediction models.

## Methods

### Participants

**Fig. 1:**
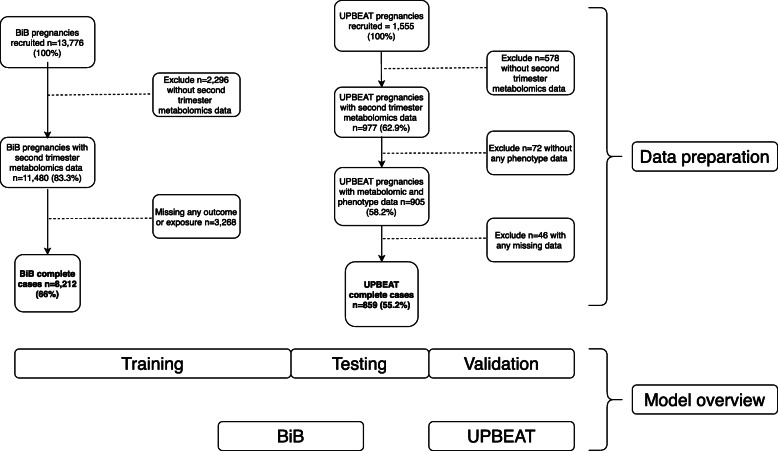
The flow of participants eligible for this study. Data preparation: flow of participants (above) in the Born in Bradford (BiB) cohort (top left) and UK Pregnancies Better Eating and Activity Trial (UPBEAT) randomised control trial (RCT) (top right) to generate the final sample for analysis. Model overview: sample split for model selection (below). BMI, body mass index; GDM, gestational diabetes mellitus; HDP, hypertensive disorder of pregnancy; SGA, small for gestational age; LGA, large for gestational age; PTB, preterm birth

We used data from the BiB study, a population-based prospective birth cohort that recruited 12,453 women who had 13,776 pregnancies. Full details of the study methodology were reported previously [32]. In brief, most women were recruited at their oral glucose tolerance test (OGTT) at approximately 26–28 weeks gestation, which was offered to all women booked for delivery at Bradford Royal Infirmary at the time of recruitment. Eligible women had an expected delivery between March 2007 and December 2010. Ethical approval for the study was granted by the Bradford National Health Service Research Ethics Committee (ref 06/Q1202/48). The UPBEAT study was a multicentre randomised control trial (RCT) which recruited 1555 obese pregnant women (BMI ≥ 30 kg/m^2^) between 15 and 18 + 6 weeks gestation, at eight centres across the UK [33]. UPBEAT is registered with Current Controlled Trials (ISRCTN89971375), and approvals were obtained from the UK research ethics committee (ref 09/H0802/5). Local research and development departments in participating centres approved the participation of their respective centres. All women in both studies provided written informed consent. Figure 1 illustrates the flow of participants. To be eligible for inclusion in the analysis, all women had to have a fasting pregnancy serum sample (used for NMR metabolome profiling) and information on all established risk factor predictors and all pregnancy-related disorders. This resulted in 8212 BiB women and 859 UPBEAT women being included. The participant characteristics are available in Table 1. All pregnancies were singleton pregnancies. In UPBEAT, this was by design, and in BiB, all the women with multiple pregnancies had some form of missing data and were not eligible for inclusion in the analysis. Here, we used UPBEAT as a cohort study, including both arms of the trial combined and adjusting for which arm they were allocated to. UPBEAT was an RCT looking at the effect of a tailored lifestyle intervention aimed at improving diet and physical activity [33]. The UPBEAT intervention did not influence the primary outcome of GDM or any of the pregnancy-related disorders explored here [34]. It did influence the change in several lipids, fatty acids and some amino acids from the NMR platform used here [34].

**Table 1:** The characteristics of the women in BiB and UPBEAT

Characteristic	Born in Bradford, *n* = 8212	UPBEAT, *n* = 859
Age, mean (SD)	27 (5.63)	30 (5.47)
Body mass index, mean (SD)	26.14 (5.73)	36.37 (4.98)
Smoking in pregnancy, *n* (%)	1420 (17.3)	133 (15.5)
Nulliparous, *n* (%)	3382 (41.2)	396 (46.1)
Ethnicity, *n* (%)		
*White European*	3629 (44.2)	573 (66.7)
*South Asian*	4085 (49.7)	51 (5.9)
*Caribbean/African (Black)*	152 (1.9)	164 (19.1)
*Others*	346 (4.2)	71 (8.3)
Gestational diabetes WHO, *n* (%)^a^	666 (8.1)	90 (10.5)
Gestational diabetes IADSPG, *n* (%)^b^	/	249 (29)
Hypertensive disorder of pregnancy, *n* (%)	803 (9.8)	79 (9.2)
Small for gestational age, *n* (%)	1139 (13.9)	59 (6.9)
Large for gestational age, *n* (%)	617 (7.5)	102 (11.9)
Preterm birth, *n* (%)^c^	430 (5.2)	39 (4.5)
Spontaneous preterm birth, *n* (%)	260 (3.2)	15 (1.6)

Data are expressed as mean (SD) or *n* (%) as appropriate. Data were 100% complete. Maternal age and weight/height (used to calculate body mass index (BMI)) were measured at recruitment. Smoking was defined as any smoking during pregnancy. Parity was defined as this pregnancy being their first child (nulliparous) or having previously given birth (multiparous). Ethnicity was based on self-report

^a^Gestational diabetes was diagnosed in the Born in Bradford according to the modified World Health Organization (WHO) criteria operating at the time of the study

^b^In UPBEAT, gestational diabetes was defined according to the guidelines recommended by the International Association of Diabetes and Pregnancy Study Groups (IADSPG). We conducted a sensitivity analysis using the WHO criteria in UPBEAT to check the differences were not due to different GDM criteria

^c^Preterm birth includes spontaneous and iatrogenic preterm birth (birth < 37 weeks gestation)

### Metabolomic profiling

In both studies, comprehensive metabolomic profiling was performed using high-throughput targeted NMR platform (Nightingale Health) (Helsinki, Finland) run either at the University of Bristol (BiB) or Nightingale Health (under its previous name of Brainshake) (UPBEAT) (https://nightingalehealth.com/about/technology). Of the 13,776 pregnancies in the BiB cohort, 11,476 pregnancies had a fasting serum sample taken at a single time point, between 24 and 28 weeks gestation, which was used for NMR profiling. In UPBEAT, NMR profiling was conducted at three time points during pregnancy 15–18 + 6 weeks, 27–28 + 6 weeks and 34–36 weeks gestation [33]. We used the 27–28 + 6-week time point for our main analyses because this matched the gestational age at which BiB samples were taken for NMR profiling, and as in BiB, these were fasting samples. The NMR platform quantified 156 metabolic traits common to both studies. The targeted metabolic traits measured by the platform represent a broad molecular signature of systemic metabolism including routine lipids, lipoprotein subclass profiling, fatty acid composition and several low-molecular metabolites, including amino acids, ketone bodies and gluconeogenesis-related metabolites, mostly in molar concentration units. A full list of all the traits profiled from women in both studies is provided in Additional file 1: Table S1. The NMR platform has been applied in various large-scale epidemiological studies, with detailed protocol and quality control information being previously published [35, 36].

### Maternal pregnancy measurements

For all outcomes, we compared the predictive ability of the metabolomic measures in relation to a set of common predictors that are routinely used in antenatal care to risk stratify women: maternal age, early pregnancy/recruitment BMI, parity, ethnicity and smoking during pregnancy. This information was collected during recruitment or extracted from clinical records in both studies. In both studies, data on parity were extracted from the first antenatal clinic records (around 12 weeks of gestation) and categorised as having experienced one or more previous pregnancy ≥ 24 weeks gestation or no previous pregnancy. Ethnicity was self-reported or obtained from primary care medical records and was categorised using the UK Office of National Statistics criteria: (1) White European (‘White British’ or ‘White European’), (2) South Asian (‘Pakistani’, ‘Indian’ or ‘Bangladeshi’), (3) Caribbean or African (‘Afro-Caribbean’ or ‘African’) or (4) others. Information on maternal age and smoking was obtained at recruitment (24–28 weeks gestation in BiB and 15–18 + 6 weeks in UPBEAT) via a researcher interview. Smoking was dichotomised as any smoking during pregnancy. In BiB, weight was extracted from the first antenatal clinic (~ 12 weeks) and height measured at recruitment. In UPBEAT, weight and height were measured at recruitment (15–18 + 6 weeks).

We examined the predictive discrimination for five pregnancy-related disorders: GDM, HDP, SGA, LGA and PTB. In BiB, all blood pressure measures and proteinuria measurements taken at any time during pregnancy were extracted from medical records [1]. In UPBEAT, these measures were taken at the participating centres and the diagnoses reported in the main centre. In both studies, gestational hypertension was defined as a new onset of elevated blood pressure (systolic blood pressure > 140 mmHg or greater and/or diastolic blood pressure > 90 mmHg or greater) after 20 weeks of gestation on two or more occasions. PE was defined as > 1 or greater ‘+’ on the reagent strip reading (equivalent to 30 mg/100 mL/mmol) or greater > 30 mg/mmol or greater on spot urine protein/creatinine ratio. We a priori decided that there were too few cases in BiB to examine the prediction of PE separately from gestational hypertension, so we combined these to generate the ‘hypertensive disorder of pregnancy’ variable used in this study. As the criteria for HDP require at least two high blood pressure measurements after 20 weeks gestation and the fasting blood samples that were used for NMR metabolite analyses were taken at 26–28 weeks, it is possible some women will have met the criteria for HDP before metabolite assessment. We explored whether the inclusion of these women influenced our main results in a sensitivity analysis in BiB by excluding any women that we defined as a HDP case before metabolite analyses (see Sensitivity analysis). All women in BiB and UPBEAT were offered a 75-g OGTT at 27–28 weeks of gestation. In BiB, fasting and 2 h post-load samples were analysed; in UPBEAT, fasting, 1 h and 2 h glucose were analysed. In BiB, GDM was defined according to the modified World Health Organization (WHO) criteria operating at the time of the study (fasting glucose ≥ 6.1 mmol/L or 2 h post-load glucose ≥ 7.8 mmol/L^3^). All the GDM cases in BiB were diagnosed at the same time as the NMR samples were taken at the OGTT. Women with pre-existing type 1 or type 2 diabetes were not invited to the OGTT and instead were managed by an endocrinologist throughout the pregnancy. Thus, none of the women with NMR samples was known to have diabetes at the time of blood sampling. In UPBEAT, GDM was defined according to the guidelines recommended by the International Association of Diabetes and Pregnancy Study Groups (IADPSG) (fasting glucose ≥ 5.1 mmol/L, 1 h glucose ≥ 10.0 mmol/L or higher, 2 h venous glucose of ≥ 8.5 mmol/L) [37]. All GDM cases in UPBEAT were also diagnosed at the same time as the NMR samples used in this study were taken (at the OGTT), and women with pre-existing type 1 or type 2 diabetes were excluded from the study. In both studies, the UK WHO fetal growth charts were used as the external standard for generating gestational age- and sex-standardised birthweight percentiles. SGA was defined as < 10th percentile and LGA as > 90th percentile. In both studies, PTB was defined as delivery before 37 completed weeks.

#### Statistical analysis

##### General approach

We developed three prediction models for each pregnancy-related disorder: (i) established risk factors (maternal age, early pregnancy/recruitment BMI, parity, ethnicity and smoking during pregnancy), (ii) NMR metabolites (156 metabolite traits) and (iii) combined risk factor and metabolomics predictors. Glucose was excluded from the metabolite prediction models for GDM because the samples had been taken at the OGTT and used to diagnose GDM. All three models were developed in a random subset of 75% of BiB (training set), and discrimination and calibration were assessed in the remaining 25% of BiB (testing set). External validation in UPBEAT was undertaken by assessing the performance of the models developed in the BiB training subset.

Having developed models for each outcome separately, we explored the extent to which these were consistent across outcomes based on the variables included in BiB. We also explored discrimination of models developed for one outcome with other outcomes (details in the ‘Sensitivity analysis’ section). This was done to assess the potential of having just one or a small number of models to predict all (or several) outcomes.

##### Model selection

We performed ten-fold cross-validation and penalised regression using the *caret* package in R version 3.5.1 [38]. To construct a model in the training subset using elastic net, an optimal lambda parameter must first be selected. This is done by applying ten-fold cross-validation to the training subset with a variety of lambda values. The lambda with the best cross-validated performance is then used to apply the elastic net to the training set to obtain a final predictive model. The performance of this model is internally validated by applying it to the testing subset. This process is more robust than doing just one (training and testing) analysis [39]. Penalised regression is a method for selecting which variables remain in the prediction model; variables whose coefficients are closer to the null are penalised (shrunk to zero) [40–42]. We used optimal values of alpha and lambda (weights used in penalising) that minimise residual variance and hence maximise prediction. These cross-validation analyses were undertaken in a randomly selected 75% subset of the BiB cohort, and then internal validation was performed on the remaining 25%.

##### External validation

We were unable to identify an independent study with relevant metabolomic data in a general population of pregnant women for external validation. We therefore undertook external validation in an available population of obese pregnant women (UPBEAT).

##### Assessing model discrimination and calibration for the prediction of pregnancy outcomes

We assessed model discrimination using AUC, ranging from no discriminative ability (0.5) to perfect discriminative ability (1). We assessed calibration (the extent to which our model predicted the probability of outcomes matched observed risk) using calibration slopes.

##### Sensitivity analysis

To explore whether the different definition of GDM in BiB and UPBEAT influenced the results, we estimated the AUC for our GDM model using the same OGTT criteria as those applied to BiB. We used the individual glucose measurements from the women in the UPBEAT study to define as GDM using the criteria (fasting glucose ≥ 6.1 mmol/L or 2 h post-load glucose ≥ 7.8 mmol/L).

As our external validation sample was only in obese pregnant women, we were concerned that any failure to validate might be due to the differences in BMI distribution between BiB and UPBEAT. To explore this, we compared the association of BMI with the five pregnancy-related disorders in (1) BiB, (2) women in BiB with a BMI ≥ 30 kg/m^2^ and (3) UPBEAT (where all women had BMI ≥ 30 kg/m^2^). This enabled us to determine whether BMI relates differently to the outcome when only obese women are included.

To evaluate whether we could use one model to predict more than one pregnancy-related disorder, we estimated the AUC for the other outcomes using the models trained and tested in BiB that had an AUC ≥ 0.6 for their specific outcome (e.g. we estimated the AUC for predicting HDP, SGA and LGA using the GDM models).

We repeated all analyses in BiB and UPBEAT for ‘any’ pregnancy-related disorder (i.e. comparing those with any GDM, HDP, SGA, LGA or PTB to those with none).

Some of the women potentially met the diagnostic criteria for HDP before blood sampling for the metabolomic analyses. Based on previous research, we believe this to be a small number [1]. To explore whether including these women in our main analyses influenced our findings, we identified and removed women meeting the HDP criteria prior to metabolomic analyses.

In the main analyses, we used the 27–28 + 6-week UPBEAT time point for the validation to match the timing of our discovery sample. In the sensitivity analyses, we explored the correlations of the 27–28 + 6-week measures with those undertaken in UPBEAT using the earlier time point (15–18 + 6 weeks gestation) of metabolite measurements and repeated all UPBEAT validation analyses. There were several reasons for this: (i) the later measures in UPBEAT were post-randomisation and could have been influenced by the intervention; (ii) the earlier measures were exploring prediction with all outcomes occurring after NMR analyses, compared with the later measures (used in BiB and UPBEAT) which are at the same time as GDM diagnoses and later than some of the HDP diagnose; (iii) the earlier measures explored prediction of GDM as opposed to metabolites associations with GDM diagnosis, and also before HDP diagnosis; and (iii) earlier antenatal prediction would enable earlier intervention to reduce risk and provide targeted treatment.

We examined prediction of spontaneous PTB, defined as those who had given birth before 37 weeks, with natural onset of labour (no medical or surgical induction). As there were only 15 spontaneous PTB in UPBEAT, we did not seek to replicate the model that was trained and tested in BiB (*n* = 260 spontaneous preterm).

## Results

Distributions of age, smoking, parity, HDP and PTB were broadly similar between the two cohorts. Differences in ethnicity reflected the sampling frame for each study. Other notable differences reflected the selection of only obese women in UPBEAT. They had a higher mean BMI and higher prevalence of GDM and LGA but lower prevalence of SGA. The higher prevalence of GDM also reflects the different diagnostic criteria used in the two studies. Proportions remained higher in UPBEAT when the same criteria used in BiB were applied, but with a smaller difference between the two studies.

### Variables included in the final models for each outcome and overlap between these

Table 2 shows the number of predictors retained in each model during model training in BiB. A full list of the predictors retained in any of the prediction models can be found in Additional file 1: Tables S2-S4.

**Table 2:** Number of predictors retained in each model developed and tested in Born in Bradford from total possible (*n* (%)). Percentages are rounded to the nearest whole number

Outcome	Model (retained predictors/total number of variables possible [%])
Gestational diabetes mellitus	Risk factor (5/5 [100%])
	Metabolite (140/156 [90%])
	Combined (152/161 [94%])
Hypertensive disorder of pregnancy	Risk factor (4/5 [80%])
	Metabolite (50/156 [32%])
	Combined (38/161 [24%])
Small for gestational age	Risk factor (4/5 [80%])
	Metabolite (86/156 [55%])
	Combined (101/161 [63%])
Large for gestational age	Risk factor (4/5 [80%])
	Metabolite (65/156 [42%])
	Combined (56/161 [35%])
Preterm birth	Risk factor (4/5 [80%])
	Metabolite (19/156 [12%])
	Combined (18/161 [11%])

Of the total 161 variables included in the combined risk factor and metabolites model, most (94%) were retained in the GDM model and least (11%) in the PTB model. At least 4, of the 5 established risk factors were retained in the combined risk factor and metabolite models for all outcomes, including BMI, parity, smoking and ethnicity. Only for PTB was age not retained. We found little overlap between predictors retained in the combined risk factor and metabolite models across outcomes. Only ten predictors were common across all combined risk factor and metabolite models (Additional file 1: Table S5). These were BMI, parity, smoking, ethnicity, creatinine, phenylalanine, isoleucine, glycine, valine and pyruvate. Very low-density lipoproteins (VLDL) (ranging from extra-large to small) were retained predictors in the prediction models for GDM, HDP, SGA and LGA. Monounsaturated fatty acids (MUFA), ratios of MUFA and omega 3 fatty acids to total fatty acids and a ratio of apolipoprotein B to apolipoprotein A-1 (APOA:APOB1) were retained predictors for GDM and LGA.

### Model discrimination and calibration

**Fig. 2:**
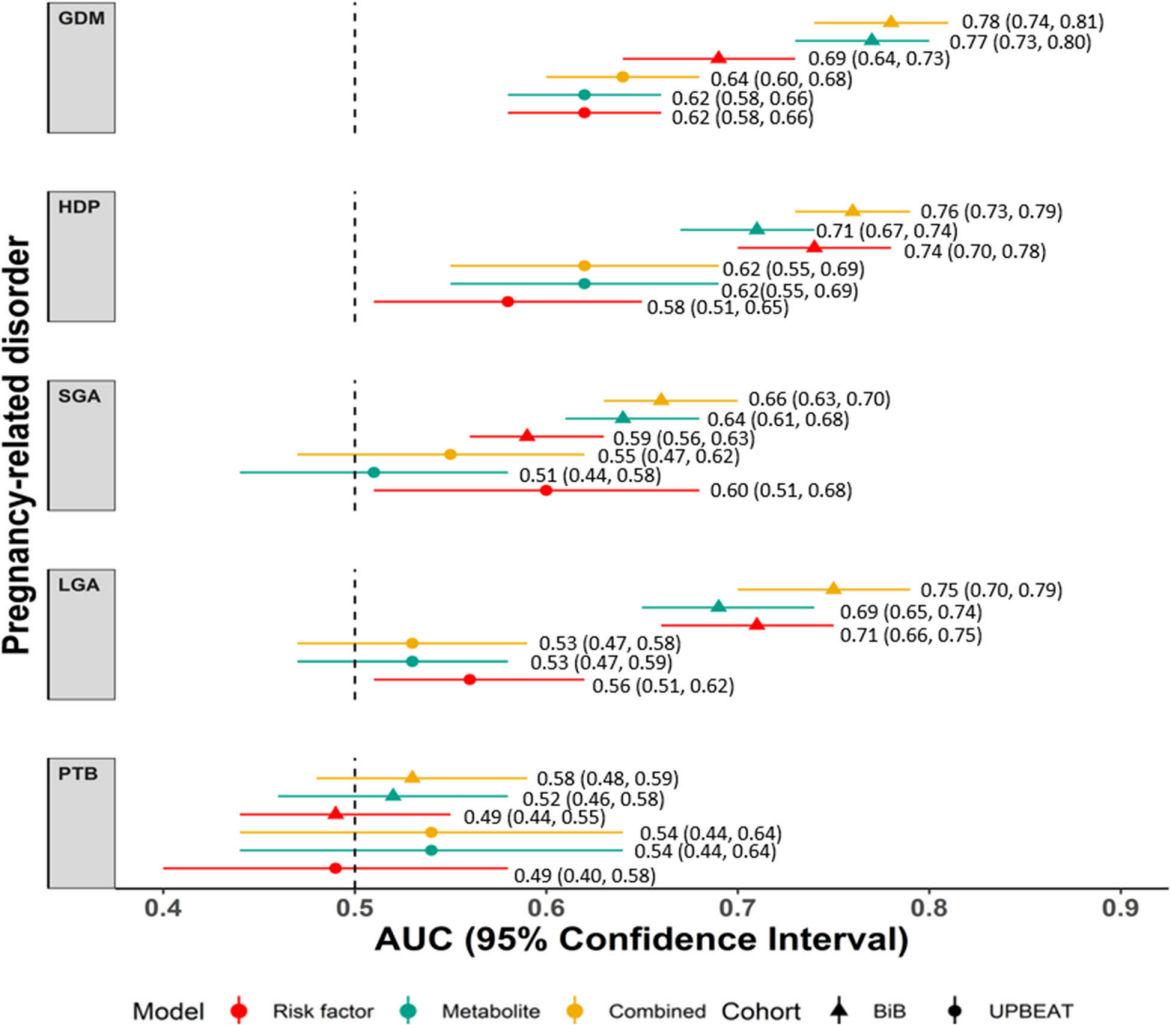
The area under the curve (AUC) for all three models with all outcomes in the Born in Bradford (triangles) and the UK Pregnancies Better Eating and Activity Trial (circles). Predictive discrimination of models for each outcome: AUC and 95% confidence intervals are shown for established risk factor prediction models (red), metabolite models (green) and combined risk factor and metabolite models (yellow) in BiB (triangles) and UPBEAT (circles). GDM, gestational diabetes; HDP, hypertensive disorders of pregnancy; SGA, small for gestational age; LGA, large for gestational age; PTB, preterm birth (iatrogenic or spontaneous) (Additional file 1: Table S6)

In BiB, discrimination for GDM, HDP and LGA was good (Fig. 2, range of AUC for all models across these three outcomes 0.69 to 0.78) for all models and improved with the addition of metabolites to the risk factors only model, particularly for GDM (difference in AUC (95% CI) 0.09 (0.08, 0.10), 0.02 (0.03, 0.01) and 0.04 (0.04, 0.03) respectively for GDM, HDP and LGA). Modest discrimination for the SGA risk factor-only model (AUC (95% CI) 0.59 (0.56–0.63)) improved when metabolites were added (AUC (95% CI) 0.66 (0.63, 0.70)). For PTB, discrimination was poor in all models (AUC ~ 0.5).

**Fig. 3:**
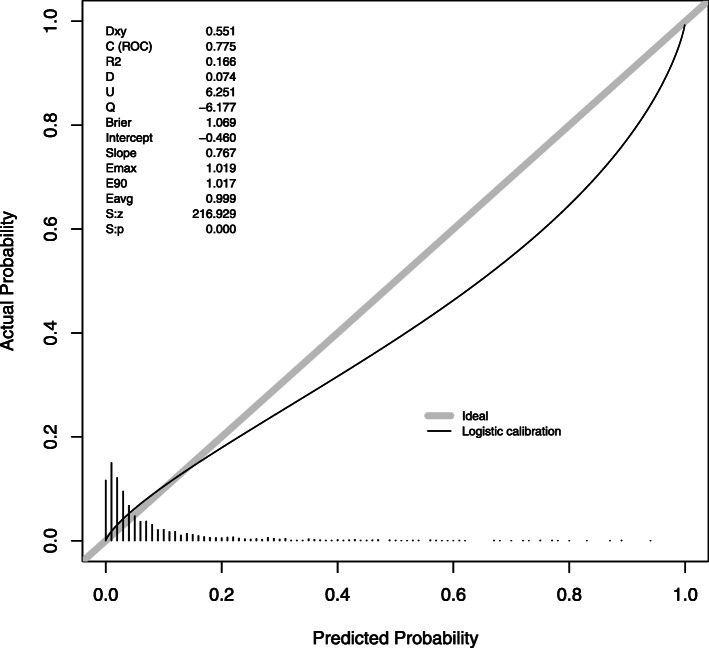
The calibration slope for the combined risk factor and metabolite model in Born in Bradford for gestational diabetes mellitus, as diagnosed using the World Health Organization criteria

**Fig. 4:**
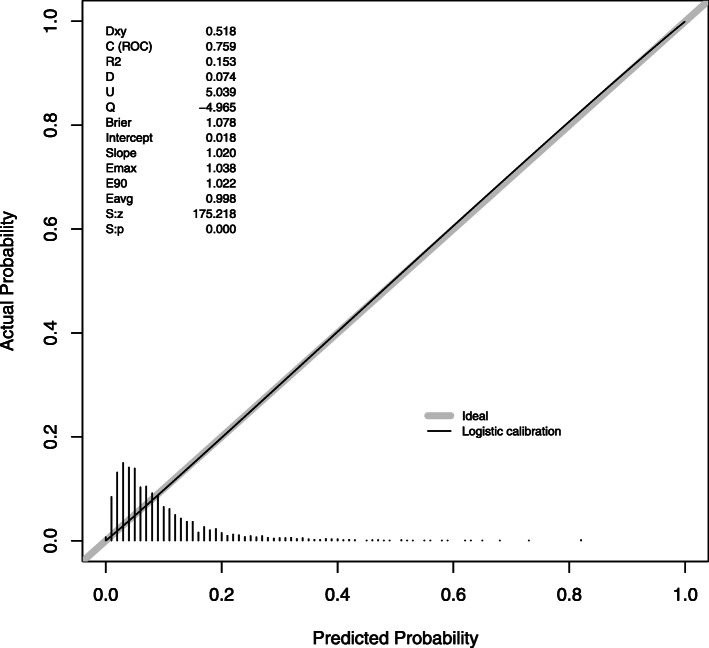
The calibration slope for the combined risk factor and metabolite model in Born in Bradford for hypertensive disorders of pregnancy

**Fig. 5:**
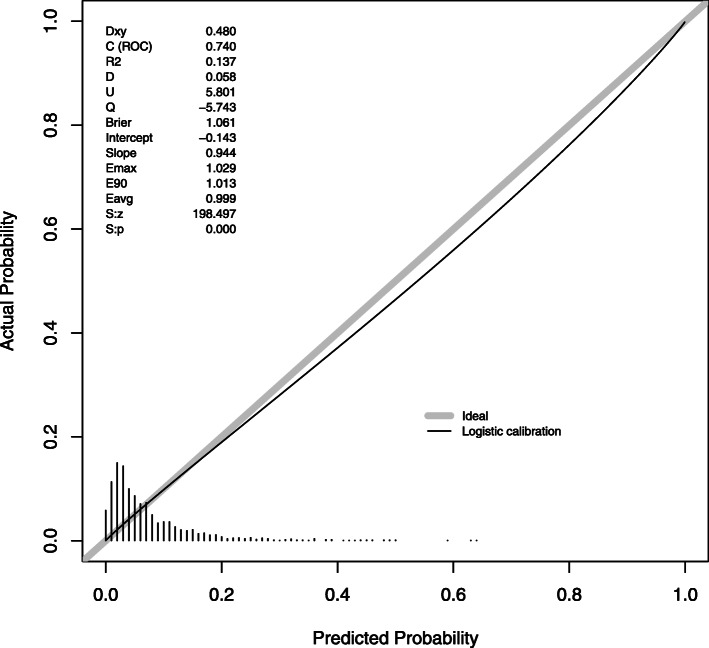
The calibration slope for the combined risk factor and metabolite model in Born in Bradford for large for gestational age

We evaluated calibration of the models which had performed well: GDM, HDP and LGA in BiB (Figs. 3, 4 and 5). As the intercepts on the slopes show, calibration is good for GDM and LGA, but with some overestimation of GDM and underestimation of LGA compared with the observed incidence. The combined risk factor and metabolite model for HDP had the best calibration.

### External validation

External validation in UPBEAT revealed similar patterns of results to those in BiB (Fig. 2). AUC was higher for the GDM and HDP combined risk factor and metabolite models when compared to the risk factor models. However, across all models, we saw lower discrimination (AUC lower by ~ 1). For example, the combined risk factor and metabolite model AUC (95% CI) for GDM was 0.78 (0.74, 0.81) in BiB and 0.62 (0.56, 0.69) in UPBEAT. Equivalent results for HDP were AUC (95% CI) 0.76 (0.73, 0.79) in BiB and 0.62 (0.55, 0.69) in UPBEAT.

### Sensitivity analysis

We did not find that criteria used to diagnose GDM significantly impacted upon the results. The combined risk factor and metabolite model for the UPBEAT GDM models using the IADSPG criteria was AUC (95% CI) 0.64 (0.60, 0.68). Using the WHO criteria, as in BiB, the combined risk factor and metabolite model discrimination had an AUC (95% CI) of 0.65 (0.58, 0.71) (Additional file 1: Table S6).

The strength and direction of the association between BMI and each outcome were similar in the whole BiB cohort and the BiB cohort including only obese women; associations in UPBEAT were weaker than either BiB dataset (Additional file 1: Table S7).

To assess the possibility that one predictive model could predict more than one outcome, we evaluated the discrimination of models developed for outcomes for which they were not trained. None of the models performed as well when applied to different outcomes (Additional file 1: Table S8).

Of the 8212 women in the BiB analysis, 2805 had ‘any’ of GDM, HDP, SGA, LGA or PTB. The risk factor models had poor discrimination in BiB (AUC (95% CI) 0.60 (0.57, 0.62)) and in UPBEAT (AUC (95% CI) 0.57 (0.53, 0.61)), only slightly improving in the combined risk factor and metabolite models: BiB (AUC (95% CI) 0.63 (0.60, 0.66)) and in UPBEAT (AUC (95% CI) 0.62 (0.60, 0.66)).

Of the 803 women meeting the HDP diagnostic criteria in BiB, 72 (9%) met these criteria prior to metabolite analyses. We removed these women and reran the analyses with the remaining 8150 women. The results did not differ notably when these 72 women were removed from the analyses: AUC (95% CI) of comparing results with these women removed to the main analyses were 0.73 (0.69, 0.76) versus 0.76 (0.73, 0.79) for the combined model, 0.71 (0.68, 0.75) versus 0.71 (0.67, 0.74) for the metabolite model and 0.71 (0.67, 0.75) versus 0.74 (0.70, 0.78) for the risk factor model.

Performances of models in UPBEAT were similar when applied to NMR metabolites obtained from the 15-18+6 week samples (Additional file 1: Table S9). The combined risk factor and metabolite model AUC was the same for HDP (AUC 0.62) at both time points. The combined risk factor and metabolite model AUC was similar for GDM (AUC (95% CI) 0.62 (0.57, 0.66) and 0.65 (0.60, 0.69) at 15 and 27 weeks, respectively), LGA (AUC (95% CI) 0.52 (0.45, 0.59) and 0.57 (0.51, 0.63)), SGA (AUC (95% CI) 0.51 (0.43, 0.59) and 0.55 (0.47, 0.62)), and PTB (AUC (95% CI) 0.52 (0.42, 0.62) and 0.54 (0.44, 0.64)). There was a good correlation between the measures at the two time points (mean correlation 0.68) (Additional file 1: Table S10).

When we trained and tested the models for spontaneous PTB in BiB, we obtained a combined risk factor and metabolite model for spontaneous PTB that had better discrimination than any (iatrogenic or spontaneous) PTB. The combined risk factor and metabolite model AUC (95% CI) for spontaneous PTB was 0.58 (0.51, 0.65) compared to AUC (95% CI) 0.53 (0.48, 0.59) for any PTB. However, the risk factor-only model had the highest AUC (95% CI) at 0.65 (0.57, 0.72), with the metabolite-only model performing poorly (AUC (95% CI) 0.48 (0.42, 0.56)) (Additional file 1: Table S6).

## Discussion

Using data from BiB, a large multi-ethnic cohort, we have shown good discrimination and calibration for GDM, HDP and LGA can be obtained from a combination of established risk factors and metabolites. The overall pattern of discrimination results was validated in a smaller independent cohort of obese pregnant women, though the AUCs were weaker. These findings show promise for the use of NMR-derived metabolites to improve prediction of common pregnancy complications, though we acknowledge the need to undertake further validation in a large independent sample of unselected women. To date, we have not been able to find such a study.

The proportion of GDM was more than three times greater in UPBEAT compared with BiB when you used the IADSPG criteria. The proportion was more similar, but still higher (10.3% in UPBEAT compared to 8.1% in BiB) when using the WHO criteria. The lower proportion of those who are SGA and the higher proportion of those who are LGA in UPBEAT is are also likely to reflect the fact that UPBEAT includes only obese women. The prevalence of HDP and PTB was similar between the two cohorts.

We found little overlap in the predictors retained in models for each outcome. Risk factors (maternal age, pregnancy smoking, BMI, ethnicity and parity) were retained in the combined risk factor and metabolite models for all pregnancy-related disorders (aside from age for PTB), demonstrating their importance in clinical prediction. Only ten predictors were retained for all pregnancy-related disorders in the combined risk factor and metabolite models (BMI, parity, smoking, ethnicity, creatinine, phenylalanine, isoleucine, glycine, valine and pyruvate). VLDL (ranging from extra-large to small) were retained predictors in GDM, HDP, SGA and LGA combined risk factor and metabolite models. Triglycerides in chylomicrons and extremely large VLDL were retained in combined risk factor and metabolite models of HDP, SGA and LGA and cholesterol esters in chylomicrons, and extremely large VLDL were retained in the GDM models. MUFA, APOA:APOB1 and a ratio of MUFA and omega-3 fatty acids to total fatty acids (%) were retained in the GDM and LGA combined risk factor and metabolite models. These results suggest that lipid monitoring might be important in discriminating between those at high risk of pregnancy-related disorders. However, it is important to note that the NMR metabolomic platform, as opposed to other metabolomic platforms, e.g. like mass spectrometry, is largely made up of lipoproteins and fatty acids (which are all part of the lipidome). The lack of overlap between metabolites retained in the models, and the poor performance of the ‘any’ pregnancy-related disorder model suggests it is unlikely one metabolite will predict pregnancy complications, and networks of metabolites are more useful for the prediction of pregnancy-related disorders.

The overall best discrimination was seen for the combined (established risk factors and metabolite) models for predicting GDM, HDP and LGA. Discrimination for GDM with the combined risk factor and metabolite model (AUC 0.78) was similar to that previously reported for GDM prediction based on clinical information, such as previous history of GDM or LGA and sociodemographic characteristics (AUC ~ 0.78) [43]. The model performs better than a previously reported model of risk factor variables (age, previous GDM, family history of type 2 diabetes, systolic blood pressure, skinfold thicknesses and waist to height/neck to thigh ratios (AUC 0.71)). This risk factor model improved when it included biomarkers such as glucose, adiponectin, sex hormone-binding globulin and triglycerides were included (AUC 0.77), but not with the addition of NMR metabolites (AUC 0.77) [29]. However, our combined risk factor and metabolite model has the advantage in that it can be applied to nulliparous women and does not rely on personal and family medical history. The combined risk factor and metabolite models for GDM, HDP and LGA in our study had good discrimination and calibration. One aim of this study was to explore the extent to which a group of potential predictors (metabolites or established risk factors) might predict several pregnancy outcomes. However, the best performing models (combined risk factor and metabolite models for GDM, LGA and HDP) showed only modest discrimination for other outcomes (AUC ranging from 0.60–0.68), with the strongest being for the prediction of LGA using the GDM combined risk factor and metabolite model (Additional file 1: Table S8). Overall, these findings for the NMR metabolite platform suggest that it may not be possible to develop a single prediction model that is accurate for several adverse pregnancy outcomes.

For HDP and SGA, whilst the combined risk factor and metabolite models had good discrimination, the metabolites did not substantially improve the discrimination or calibration when compared to the established risk factors. In the interests of maximising the sample, our HDP variable included both gestational hypertension and PE, and our model discrimination for HDP was weaker than that reported for the sFlt-1:PlGF ratio for PE alone [24] and that observed for a model including first antenatal clinical characteristics and repeat antenatal blood pressure measurements for PE or gestational hypertension alone (AUC 0.77–0.88) [6]. It would be useful to repeat our analyses in a larger study with sufficient power to explore the predictive ability of metabolites for PE and gestational hypertension separately.

Previous studies have reported better discrimination for SGA using metabolite models than reported in this study. However, sample sizes were small and there was no external validation or assessment of calibration [25, 26]. We used a < 10% cut-off for SGA, as recommended by the WHO. Some recommendations advise using a more conservative < 3% cut-off [28], whilst there is also evidence that a threshold of 25% better predicts stillbirth and neonatal mortality [44]. We lacked power in this study to explore a range of different thresholds for SGA and LGA and be able to precisely detect differences between them.

For any PTB (iatrogenic or spontaneous), discrimination was very poor across all models. When the analyses were limited to spontaneous PTB, the discrimination for all models was higher than for the models with any PTB. However, the AUC remained poor for the combined (AUC (95% CI) 0.58 (0.51, 0.65)) and metabolite-alone model (AUC (95% CI) 0.48 (0.41, 0.56)), with modest discrimination for the risk factor model (AUC (95% CI) 0.65 (0.57, 0.72)). We acknowledge that because of its aetiological presentation, spontaneous PTB is likely to be difficult to predict. These results highlight the need for better models to predict PTB, or its subtypes, aside from a previous history of PTB. Our results also suggest that metabolomics quantified with the targeted NMR platform used here are not useful for predicting iatrogenic or spontaneous PTB.

The low discrimination observed when predicting ‘any’ pregnancy-related disorder (GDM, HDP, SGA, LGA or PTB) was unexpected given that similar risk factors are predictive of each disorder. However, this is consistent with the poor performance of models trained for one pregnancy-related disorder but applied to others. It suggests that the biological basis of each disorder is more complex than indicated by a small number of risk factors.

We were unable to identify a general population of pregnant women with relevant data for validation, so we performed validation in obese pregnant women (UPBEAT). In this sample, models demonstrated poorer discrimination. It is generally expected that prediction is poorer in external validation samples [45], but it is also likely that this has also been influenced by the different incidences of some outcomes between the two cohorts and the distinct metabolic perturbations experienced by obese women during pregnancy [23]. Further studies exploring the value of metabolomic analysis during pregnancy are needed [46].

### Strengths and limitations

Previous studies aiming to improve prediction of pregnancy-related disorders often do not compare performance to established risk factors, assess calibration or undertake external validation as undertaken here [47–50]. A strength of this study was the greater number of women with NMR data in BiB compared to previous studies of metabolite prediction. The NMR platform has several strengths in relation to its use for prediction; measurements are reliable with little variation between batches, the volume of plasma or serum required for analyses is small (100–300 μL) and to obtain all measures is not expensive (~ £20) [51]. NMR provides absolute quantification, which represents clinically useful units. However, the platform quantifies only a small proportion of the metabolome. Other platforms, such as mass spectrometry, quantify over 1000 metabolites [52]. With greater coverage of the metabolome, it is possible that we would have improved prediction for the pregnancy outcomes explored here. We were limited in this study by the BiB NMR samples being taken in the second trimester (samples taken for the OGTT). This meant that the GDM cases were diagnosed at the same time as the metabolite samples, and some of the HDP cases met the criteria for this diagnosis before metabolite assessment. When we removed the 72 women who met the HDP criteria before the metabolite assessment, the results were very similar to those in our main analysis. Furthermore, when we performed the validation using the 15–18 + 6-week gestation data from UPBEAT, the results were comparable to the second trimester results in UPBEAT (Additional file 1: Table S9) and metabolites at 26 weeks correlated with those at 15 weeks (Additional file 1: Table S10). Taken together, these suggest that the metabolites measured in the second trimester are good proxies for earlier antenatal measures of the same metabolites. However, this needs to be directly tested. Ideally, we would have a prediction tool that could be used as early as possible in pregnancy. It would be able to be repeated so that women’s antenatal care could be tailored to their risk from early pregnancy and updated with repeat assessment if risk changed. In addition to UPBEAT including only obese pregnant women, the cohort differed from BiB in including singleton pregnancies only. Multiple pregnancies from BiB were not excluded from BiB by design, but after removing those with missing data, only singleton pregnancies remained.

## Conclusions

To conclude, our results suggest metabolomics combined with established risk factors improve prediction of GDM, HDP, LGA and SGA, compared to established risk factors alone. As we were only able to explore validation in a select cohort of obese women, these findings should be validated in large, general cohorts of pregnant women. A predictive test for all or several of these outcomes would have significant clinical importance and allow us to identify mothers in need of further resources and antenatal monitoring. However, we found relatively little overlap in the models for different outcomes and poor discrimination for other outcomes for any combined risk factor and metabolite model than the outcome it had been developed for. By improving the allocation of resources and stratifying antenatal care from early pregnancy until delivery, we can reduce the burden on the healthcare providers and the morbidity and mortality of mothers and offspring.

## Supplementary Information

**Additional file 1: Table S1.** A list of the nuclear magnetic resonance (NMR)-derived metabolomic traits used in this study. **Table S2.** The retained predictors for the risk factor prediction models for all pregnancy-related disorders. **Table S3.** The retained predictors for the metabolomic prediction models for all pregnancy-related disorders. **Table S4.** The retained predictors for the combined risk factor and metabolomic prediction models for all pregnancy-related disorders. **Table S5.** The predictors that were retained in the combined risk factor and metabolite model for all pregnancy-related disorders. **Table S6.** The area under the curve and 95% confidence intervals for each prediction model for each pregnancy-related disorder. **Table S7.** A sensitivity analysis on the association between body mass index (BMI) and each pregnancy-related disorder in the UK Better Eating and Activity Trial (UPBEAT), the Born in Bradford study and the Born in Bradford study women who had a BMI ≥30 kg/m2. **Table S8.** The area under the curve and 95% confidence intervals for prediction models trained in one pregnancy-related disorder and tested for another pregnancy-related disorder. **Table S9.** The area under the curve and 95% confidence intervals for prediction models trained in Born in Bradford and validated at an earlier timepoint in the UK Pregnancies Better Eating and Activity Trial. **Table S10.** The correlation between metabolites measured in the UK Pregnancies Better Eating and Activity Trial at 15-18+6 weeks gestation and 27-28+8 weeks gestation.

## Data Availability

Data is available upon request from Born in Bradford https://borninbradford.nhs.uk/research/how-to-access-data/ and the UK Pregnancies Better Eating and Activity Trial https://www.medscinet.net/upbeat/.

## Abbreviations

AUC: Area under the curve
BiB: Born in Bradford
BMI: Body mass index
GDM: Gestational diabetes mellitus
HDP: Hypertensive disorders of pregnancy
LGA: Large for gestational age
NMR: Nuclear magnetic resonance
PTB: Preterm birth
SGA: Small for gestational age
UPBEAT: UK Pregnancies Better Eating and Activity Trial
